# Pentameric assembly of the Kv2.1 tetramerization domain

**DOI:** 10.1107/S205979832200568X

**Published:** 2022-05-30

**Authors:** Zhen Xu, Saif Khan, Nicholas J. Schnicker, Sheila Baker

**Affiliations:** a Protein and Crystallography Facility, University of Iowa, 51 Newton Road, Iowa City, IA 52242, USA; b Department of Biochemistry and Molecular Biology, University of Iowa, 51 Newton Road, Iowa City, IA 52242, USA

**Keywords:** voltage-gated potassium channels, Kv2.1, tetramerization domain, crystal structure, SAXS, pentamer, tetramer

## Abstract

The Kv family of voltage-gated potassium channels regulate neuronal excitability. The biophysical characteristics of Kv channels can be matched to the needs of different neurons by forming homotetrameric or heterotetrameric channels within one of four subfamilies. The cytoplasmic tetramerization (T1) domain plays a major role in dictating the compatibility of different Kv subunits. The only Kv subfamily lacking a representative structure of the T1 domain is the Kv2 family. Here, X-ray crystallography was used to solve the structure of the human Kv2.1 T1 domain. The structure is similar to those of other T1 domains, but surprisingly formed a pentamer instead of a tetramer. In solution the Kv2.1 T1 domain also formed a pentamer, as determined by inline SEC–MALS–SAXS and negative-stain electron microscopy. The Kv2.1 T1–T1 interface involves electrostatic interactions, including a salt bridge formed by the negative charges in a previously described CDD motif, and inter-subunit coordination of zinc. It is shown that zinc binding is important for stability. In conclusion, the Kv2.1 T1 domain behaves differently from the other Kv T1 domains, which may reflect the versatility of Kv2.1, which can assemble with the regulatory KvS subunits and scaffold ER–plasma membrane contacts.

## Abstract 2

The selective assembly of Kv subunits into one of four subfamilies of tetrameric, voltage-gated potassium channels is mediated by the T1 tetramerization domain. Here, it was found that unlike the other Kv T1 domains that have been studied to date, the human Kv2.1 T1 domain forms a pentamer, and that zinc binding and electrostatics contribute to the stability of the proteins.

## 1. Introduction

Voltage-gated potassium channels (Kvs) are essential for regulating membrane potential, propagating action potentials and controlling potassium homeostasis in a diverse array of neuronal and non-neuronal tissues (Allen *et al.*, 2020 ▸; Wulff *et al.*, 2009 ▸). There are 27 Kv genes in the human genome, which are classified into five subfamilies with distinct expression patterns and biophysical properties: Kv1 (Shaker), Kv2 (Shab), Kv3 (Shaw), Kv4 (Shal) and the regulatory KvS (Silent). Functional diversity is achieved by mixing and matching Kv monomers to form heterotetrameric channels in addition to homotetrameric channels (Isacoff *et al.*, 1990 ▸). Only Kv subunits within the same subfamily can interact. The KvS proteins are an exception to this: they do not form homomeric channels, but instead must assemble with Kv2 subunits (Ottschytsch *et al.*, 2002 ▸; Bocksteins, 2016 ▸). The major constraint on inter-subfamily assembly is the cytoplasmic tetramerization (T1) domain (Li *et al.*, 1992 ▸; Shen & Pfaffinger, 1995 ▸; Xu *et al.*, 1995 ▸; Shen *et al.*, 1993 ▸). How the T1 domain confers selective assembly is poorly understood.

The T1 domain is one of four structural classes in the BTB (or POZ) superfamily of protein–protein interaction domains (Stogios *et al.*, 2005 ▸). The core BTB fold comprises ∼95 amino acids and is comprised of five α-helices organized into two sets of hairpins that are capped at one end by three β-strands. Deletions, insertions or extensions in the core BTB fold characterize different structural classes, with the T1 class from Kv proteins being the most similar to the core BTB fold. BTB folds are most often found as homodimers or, in the case of T1 domains, tetramers. One interesting exception is the BTB fold from KCTD proteins, which may form monomers, dimers, tetramers, pentamers or even hexamers *in vitro*, independent of their stoichiometry in native protein complexes (Pinkas *et al.*, 2017 ▸; Sereikaite *et al.*, 2019 ▸).

While there are no representative T1 structures from either the Kv2 or KvS subfamilies, structural analysis of T1 domains from Kv1, Kv3 and Kv4 proteins have provided key insights into how the selective assembly of tetramers occurs. Some subfamily-specific individual differences in charged or hydrophobic amino acids facing the tetramerization interface have been identified as important for assembly (Bixby *et al.*, 1999 ▸; Shen & Pfaffinger, 1995 ▸; Kreusch *et al.*, 1998 ▸; Nanao *et al.*, 2003 ▸; Stogios *et al.*, 2005 ▸). Another critical factor in selective T1-domain assembly is inter-subunit coordination of zinc. An H*X*_5_C*X*_20_CC motif is conserved in Kv2, Kv3 and Kv4 proteins but is absent from Kv1 proteins (Bixby *et al.*, 1999 ▸). In Kv4.2 and Kv3.1 T1 domains Zn^2+^ binding has been shown to be essential for monomers to assemble into tetramers (Jahng *et al.*, 2002 ▸; Strang *et al.*, 2003 ▸; Nanao *et al.*, 2003 ▸). The role of Zn^2+^ binding has not been tested for Kv2 or KvS T1 domains, but it is logical to assume that it plays a similar role in their assembly. Thus, zinc-dependent assembly distinguishes the Kv1 T1 domain from the Kv2, Kv3 and Kv4 T1 domains. A feature that distinguishes Kv2 and KvS T1 domains from Kv3 and Kv4 T1 domains is the presence of a CDD motif (Bocksteins *et al.*, 2009 ▸). The authors who identified the CDD motif used the structure of Kv4.2 and *SWISS-MODEL* to generate a homology model of Kv2.1 and determined that the critical aspartates in the CDD motif are not at the T1 homotetrameric interface but are closer to the surface of the domain (Bocksteins *et al.*, 2009 ▸). This raises the possibility that interaction between the surface of Kv T1 domains and other regions of the channel could add to the subfamily-selective assembly dictated by T1-domain tetramerization. Supporting this idea is the observation that the N- and C-termini of Kv2.1 can interact (Ju *et al.*, 2003 ▸; Mohapatra *et al.*, 2008 ▸; Bocksteins *et al.*, 2009 ▸). Solving the structures of Kv2 and KvS T1 domains will be necessary steps towards the goal of completing a comprehensive comparative analysis of how T1 domains confer selective assembly.

In this study, we solved the crystal structure of the human Kv2.1 T1 domain (Kv2.1 T1) and investigated the role of Zn^2+^ in the stability of the protein. To our surprise, Kv2.1 T1 was pentameric both in the crystal and in solution. Zn^2+^, along with a salt bridge formed by the aspartates in the CDD motif, provides stability. The different assembly state of the isolated Kv2.1 T1 domain compared with the isolated T1 domains from Kv1, Kv3 and Kv4 proteins indicates that multiple interactions between Kv2.1 subunits are required *in vivo* to ensure proper assembly into tetramers.

## 2. Materials and methods

### 2.1. Cloning, overexpression and purification of Kv2.1 T1

Human Kv2.1 residues 29–147, corresponding to the T1 domain, were cloned using NdeI and BamHI sites in a modified pET-28a vector (Novagen) to express a fusion protein with an N-terminal hexahistidine (6×His) affinity tag followed by a Tobacco etch virus (TEV) protease cleavage site (ENLYFQG). Transformed *Escherichia coli* strain BL21 (DE3) cells were grown in LB medium supplemented with 50 µg ml^−1^ kanamycin at 37°C to an optical density (OD_600_) of 0.7, and 1 m*M* isopropyl β-d-1-thiogalactopyranoside was added to induce expression for 15 h at 18°C. The cells were harvested by centrifugation and stored at −80°C. The harvested cells were resuspended in lysis buffer (20 m*M* Tris–HCl pH 8.0, 150 m*M* NaCl, 5% glycerol, 0.01% Triton X-100, 20 m*M* imidazole, 5 m*M* β-mercapto­ethanol) and supplemented with DNase I and protease-inhibitor cocktail (Roche). The cells were disrupted using an Emulsiflex C3 (Avestin) to release the protein. The His-tagged fusion protein was purified using an Ni–NTA column (Qiagen) and the elution fraction was incubated with TEV protease for 15–18 h at 4°C to cleave the N-terminal His tag. The cleaved protein was further purified using a HiLoad 16/600 Superdex 200 pg gel-filtration column (GE Healthcare) in 20 m*M* Tris–HCl pH 8.0, 150 m*M* NaCl, 5% glycerol, 0.01% Triton X-100, 1 m*M* DTT. The purified protein was concentrated using Amicon Ultra centrifugal filter devices (Millipore, 10 kDa cutoff). The protein samples for dynamic light-scattering (DLS) and circular-dichroism (CD) measurements were dialyzed into phosphate buffer (20 m*M* Na_2_HPO_4_ pH 7.4, 150 m*M* NaCl, 5% glycerol, 1 m*M* DTT) at 4°C overnight before use.

### 2.2. Crystallization and X-ray diffraction data collection

Kv2.1 T1 was crystallized using the hanging-drop vapor-diffusion method by mixing 8 mg ml^−1^ Kv2.1 T1 with the reservoir solution in a 1:1 ratio. Initial crystals were obtained at 291 K in 0.2 *M* magnesium chloride, 15% PEG 400, 0.1 *M* sodium HEPES pH 7.5. The crystals were flash-cooled in liquid nitrogen using 30% PEG 400 as a cryoprotectant (see PDB entry 7spd). Crystals of Kv2.1 T1 prepared by supplementing the size-exclusion buffer with 50 µ*M* zinc sulfate were obtained in 0.2 *M* magnesium chloride hexahydrate, 0.1 *M* Tris pH 8.5, 3.4 *M* 1,6-hexanediol and were flash-cooled in liquid nitrogen without additional cryoprotection (see PDB entry 7re5). X-ray diffraction data were collected at 100 K on beamline 4.2.2 at the Advanced Light Source (ALS), Berkeley, California, USA.

### 2.3. X-ray diffraction data processing and structure refinement

Diffraction data were indexed, integrated and scaled using *XDS* (Kabsch, 2010 ▸). Initial phase estimates for Kv2.1 T1 were obtained by molecular replacement with *Phaser* in *Phenix* (Bunkóczi *et al.*, 2013 ▸) using the structure of Kv3.1 T1 (PDB entry 3kvt; Bixby *et al.*, 1999 ▸) as a search model. Refinement was performed using *phenix.refine* (Afonine *et al.*, 2012 ▸), followed iteratively by manual building using *Coot* (Emsley *et al.*, 2010 ▸). The final refined structure includes a Kv2.1 T1 pentamer with residues 29–133 resolved. Structures were visualized in *PyMOL* (version 2.5; Schrödinger). The buried surface area was determined using the *PISA* server (Krissinel & Henrick, 2007 ▸). The structural biology applications used in this project were compiled and configured by SBGrid (Morin *et al.*, 2013 ▸).

### 2.4. Size-exclusion chromatography coupled with multi-angle light scattering and small-angle X-ray scattering (SEC–MALS–SAXS)

SEC–MALS–SAXS data sets were collected on BioCAT beamline 18-ID-D at the Advanced Photon Source, Argonne, Illinois, USA. The samples were centrifuged for 5 min at 13 000 rev min^−1^ to remove any potential aggregates before injection. 250 µl aliquots containing 1.5 mg ml^−1^ Kv2.1 T1 were loaded at a flow rate of 0.5 ml min^−1^ onto a 24 ml Superdex 200 Increase 10/300 column on an Agilent 1300 chromatography system. Following elution from the column, the samples were analyzed inline by the UV absorbance detector of the Agilent 1300 chromatography system followed by DAWN HELEOS II light-scattering and OptiLab T-rEX refractive-index detectors in series. An accurate protein molecular weight was determined using the *ASTRA* software (Wyatt Technology). The elution trajectory was redirected into the SAXS sample-flow cell. Scattering data were collected every 1 s using 0.5 s exposure on a PILATUS3 1M pixel detector (Dectris) covering a *q* range of 0.0045 < *q* < 0.35 Å^−1^ (*q* = 4π/λsinθ, where λ is the wavelength and 2θ is the scattering angle). The *BioXTAS RAW* software was used to collect the SAXS data (Hopkins *et al.*, 2017 ▸).

### 2.5. Small-angle X-ray scattering (SAXS) data processing and modeling

Following data reduction and buffer subtraction, the SAXS data were further analyzed using the *BioXTAS RAW* software. The forward scattering intensity *I*(0) and the radius of gyration (*R*_g_) were calculated from the Guinier fit. The normalized Kratky plot, the pair distance distribution plot *P*(*r*) and the Porod volume were calculated using *GNOM* embedded in *BioXTAS RAW*. Low-resolution *ab initio* bead-based models of proteins were constructed from the experimental data using *GASBOR* (Svergun, 1999 ▸). The calculation of theoretical scattering curves for the crystal structure was performed by *CRYSOL* (Manalastas-Cantos *et al.*, 2021 ▸), which also determines the discrepancy (χ^2^ value) between the simulated and experimental scattering curves.

### 2.6. Circular-dichroism (CD) spectroscopy

Far-UV CD spectra of Kv2.1 T1 were acquired using a J-815 spectrometer (JASCO) connected to a Peltier temperature controller. All experiments were performed in duplicate. For each measurement, 300 µl sample consisting of 20 µ*M* Kv2.1 T1 was added to a 1 mm path-length quartz cuvette (Hellma Analytics). The CD ellipticities (θ) were measured from 200 to 260 nm with a 1 nm increment, a scanning speed of 50 nm min^−1^ and a data-integration time of 2 s with a standard sensitivity. Buffers were used for baseline measurements. The final ellipticities were recorded as an average of four baseline-corrected scans. The ellipticities (θ) were used to calculate the mean residue ellipticity using the formula [θ] = θ/*cnl*, where *c* is the concentration of the protein in moles, *n* is the number of residues and *l* is the path length of the cuvette. To examine the effect of Zn^2+^ on the stability of Kv2.1 T1, 1 m*M* EDTA was added to Kv2.1 T1 samples and incubated at 4°C for 12 h, and the far-UV CD spectra were then measured in the same way as for the samples in the absence of EDTA. To detect the thermal unfolding, denaturation experiments were carried out using an automated 1°C incremental temperature ramp in the interval 20–95°C, with a 30 s equilibration time at each measurement step. The thermal unfolding profiles of the samples were characterized using the mean residue ellipticity minimum at 222 nm (θ_222_) to determine *T*_m_ by fitting the Boltzmann sigmoid equation using *Prism* (GraphPad).

### 2.7. Dynamic light scattering (DLS)

Thermal ramp stability measurements were made by DLS in a plastic cuvette with 1 mm path length at various temperatures ranging from 10 to 80°C, with a ramp rate of 1°C min^−1^. Before measurements, the sample was incubated at 10°C for 3 min. The DLS measurements were averaged from five acquisitions of 1 s each. To examine the effect of Zn^2+^ on the stability of Kv2.1 T1, 1 m*M* EDTA was added to Kv2.1 T1 and incubated at 4°C for 12 h and the size was then measured by DLS and compared with that of samples without adding any EDTA. The DLS data were analyzed using Wyatt software and were fitted by the linear intersection method to determine the *T*_onset_ values.

### 2.8. Transmission electron microscopy

For negative staining, 3 µl protein solution at 0.01 mg ml^−1^ was added to a glow-discharged carbon-coated copper grid (Electron Microscopy Science). The grids were stained in 2%(*w*/*v*) uranyl acetate (twice for 3 s each, followed by a third staining for 25 s). The grids were blotted with filter paper (Whatman) to absorb residual solution between each step.

The grids were imaged using a Hitachi HT7800 electron microscope equipped with a tungsten filament and operated at 120 kV. Images were collected at a magnification of 100 000×, resulting in a pixel size of 1.93 A on the specimen. Only top views of particles were manually picked using *cryoSPARC* to determine the oligomeric state of Kv2.1 T1 (Punjani *et al.*, 2017 ▸). The manually picked particles were classified using 2D reference-free classification and new templates were created for automatic picking to select more particles. Two rounds of 2D reference-free classification were calculated to exclude ‘bad’ particles and were used for data analysis.

### 2.9. Kv2.1–Kv8.2 T1-domain heterotetramer model generation and zinc addition

The T1-domain sequences from Kv2.1 and Kv8.2 (in a 3:1 ratio) were submitted to the *ColabFold* notebook to generate the heterotetramer using *AlphaFold*2-*Multimer* (Mirdita *et al.*, 2022 ▸). The models were inspected and the top-ranked model was used for the addition of zinc. Potential zinc-binding sites in the predicted complex structure were identified using the method recently reported by Wehrspan *et al.* (2022 ▸). Briefly, this method seeks to superimpose a user-specified list of ligands at all possible plausible locations within a protein structure and retains all positions where a ligand can be placed free of steric clashes. The method has previously been applied to entire structural proteomes predicted using *AlphaFold*2 (Tunyasuvunakool *et al.*, 2021 ▸) and has identified thousands of potential zinc-binding sites. Here, the method was applied using the same parameters as used previously. Three zinc-binding sites were identified, all of which had r.m.s.d.s lower than 0.3 Å, indicating high-quality fits, and with all coordinating side chains having pLDDT scores exceeding 88, indicating high-confidence predictions from *AlphaFold*2 (Jumper *et al.*, 2021 ▸). The code is available at https://github.com/Elcock-Lab/Metalloproteome

## 3. Results

### 3.1. The crystal structure of the Kv2.1 tetramerization (T1) domain

**Figure 1:**
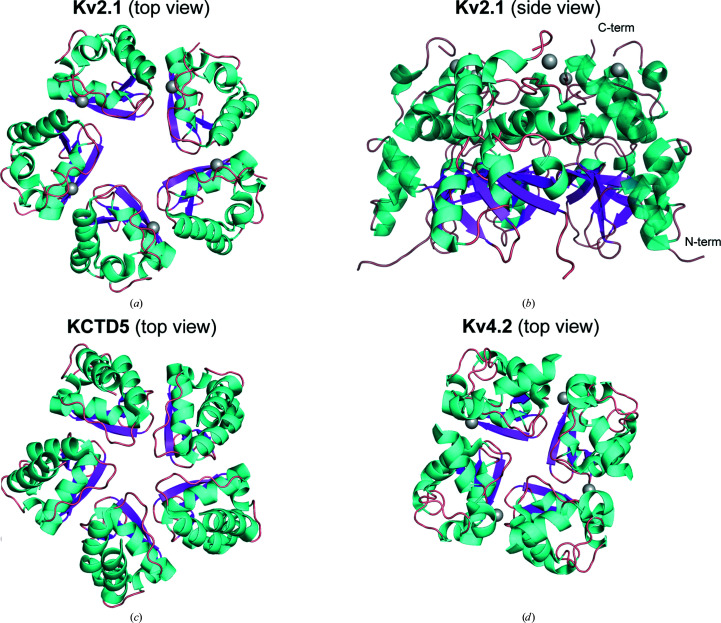
Overview of the Kv2.1 T1 crystal structure. (*a*, *b*) Cartoon representation of (*a*) the top view (C-terminus) and (*b*) the side view of the X-ray structure of Kv2.1 T1 in a pentameric form (PDB entry 7re5). (*c*, *d*) Top view of previously reported X-ray structures of (*c*) the pentamer of KCTD5 (PDB entry 3drz) and (*d*) the tetramer of Kv4.2 (PDB entry 1nn7). Colors for secondary structure are α-helices in cyan and β-sheets in magenta; the gray sphere is the zinc ion.

The T1 domain of voltage-gated potassium channel Kv2.1 (human Kv2.1 T1; residues 29–147) was expressed in *E. coli* BL21 (DE3) cells and purified by Ni–NTA chromatography followed by TEV cleavage to remove the N-terminal His tag and finally Superdex 200 chromatography. Crystals of Kv2.1 T1 belonged to space group *P*4_1_2_1_2. Initial phase estimates were obtained by molecular replacement using a Kv3.1 T1-domain monomer (PDB entry 3kvt) as a search model, and the resulting Kv2.1 T1 structure was refined at a resolution of 2.5 Å (PDB entry 7re5). Electron densities for residues 29–133 were identified; the missing C-terminal residues (134–147) may be disordered. Surprisingly, five protein molecules were present in the asymmetric unit of the Kv2.1 T1 crystals, forming a pentameric ring, as shown from the C-terminal (top) and side views (Figs. 1 ▸ *a* and 1 ▸ *b*). Diffraction data and refinement statistics are presented in Table 1 ▸. A metal ion was identified in each of the individual T1 domains, coordinated by a conserved zinc-binding H*X*_5_C*X*_20_CC motif. The Kv2.1 T1 pentameric rings were stacked as dimers in the crystal and a closer examination of the interface between rings revealed a second metal ion coordinated by residues introduced by the N-terminal NdeI cloning site. Representative electron densities and verification of the metal as Zn^2+^ by X-ray fluorescence spectroscopy are shown in Supplementary Fig. S1. We also obtained a lower resolution (2.7 Å) structure in the alternative space group *C*222_1_, which was also a pentamer but with partial zinc occupancy (for details, see PDB entry 7spd). The core BTB fold of the Kv2.1 T1 monomers is similar to the monomers of the pentameric T1-like domain from KCTD5 (Fig. 1 ▸ *c*) and the tetrameric Kv4.2 T1 domain (Fig. 1 ▸ *d*). The five Kv2.1 T1 subunits are rotated from the central axis of the ring by 70.5°, 71.3°, 71.5°, 73.2° and 73.5°, which is more open than the arrangement in KCTD5 (69.5°, 69.5°, 72.8°, 73.4° and 74.7°). Overall, the pentameric ring of Kv2.1 T1 subunits is less compact than that for KCTD5.

**Figure 2:**
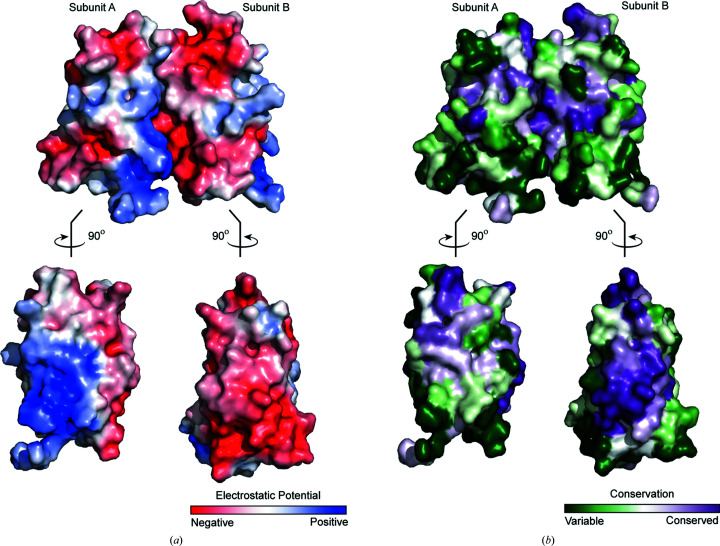
The monomer–monomer interface of Kv2.1 T1 is a conserved charged surface. (*a*) Surface representation of Kv2.1 T1 colored by electrostatic potential calculated using *APBS*. The gradient is from −5*kT*/e (red) to +5*kT*/e (blue). Top: as observed from the side view of the X-ray structure in Fig. 1 ▸(*b*). Bottom: subunits are rotated 90° with respect to the X-ray structure. (*b*) Surface representation of Kv2.1 T1 colored by sequence conservation (purple for most conserved and green for least conserved). Conservation scores were calculated using *ConSurf*. The top and bottom views are subunits rotated as described for (*a*).

The surface of the interface between adjacent Kv2.1 T1 subunits is characterized by complementary charges (Fig. 2 ▸ *a*). Electrostatics make a particular contribution to the N-terminal portion of the T1 domain, with a positively charged bulge fitting into a negatively charged concave area on the adjacent subunit. The residues constituting the negatively charged bulge are highly conserved across the T1 family. However, the residues constituting the matching positive patch are less conserved and thus may contribute to the selectivity of T1-domain assembly (Fig. 2 ▸ *b*).

**Figure 3:**
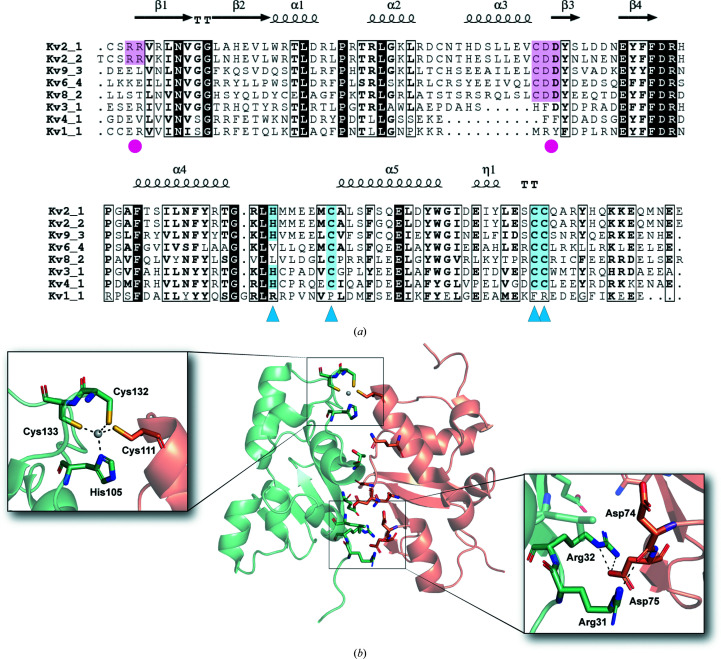
Pentamer formation involves inter-subunit zinc binding and a salt bridge. (*a*) Structure-based multiple sequence alignment of Kv2.1 with KvS (Kv9.3, Kv8.2 and Kv6.4), Kv3.1, Kv4.1 and Kv1.1 T1 domains. Blue triangles below residues indicate the Zn^2+^-coordination motif H*X*_5_C*X*_20_CC and magenta circles indicate the negatively charged CDD motif and its interacting positive residues at the N-terminus. Shading of residues in blue or magenta, respectively, highlights conservation. (*b*) Cartoon representation of Kv2.1 T1 with residues at the subunit interface highlighted as sticks; colors are chain *A* in green and chain *B* in orange. The insets show the interacting residues in the Zn^2+^-coordination site (left) or CDD motif (right). Hydrogen bonds are shown as black dashed lines, the zinc ion is in gray, O atoms are in red, S atoms are in yellow and N atoms are in dark blue.

Two motifs that have previously been implicated in selective higher-order assembly of T1 domains are the Zn^2+^ binding and CDD motifs (Fig. 3 ▸ *a*; Jahng *et al.*, 2002 ▸; Strang *et al.*, 2003 ▸; Nanao *et al.*, 2003 ▸; Bocksteins *et al.*, 2009 ▸; Bixby *et al.*, 1999 ▸). The H*X*_5_C*X*_20_CC Zn^2+^-binding motif is found in all Kv2, Kv3 and Kv4 proteins, as well as in four of the ten KvS proteins (Kv8.2, Kv6.1, Kv6.2 and Kv6.3) that assemble with Kv2 proteins. This motif, occupied by Zn^2+^, is at the C-terminal end of the T1 domain, which in the context of the full-length protein would be the end closest to the transmembrane domains. In Kv2.1 it is formed by His105, Cys132 and Cys133, with the third cysteine, Cys111, provided by the adjacent subunit (Fig. 3 ▸ *b*, inset 1). The CDD motif is found in a loop between α3 and β3 at the other end of the subunit interface from zinc (Fig. 3 ▸ *b*, inset 2). Asp74 and Asp75 of this motif are in the interface and form a salt bridge with Arg31 and Arg32. The arginines are only conserved in Kv2.1 and Kv2.2. Together, these two motifs are well positioned to provide stability to higher-ordered assemblies of Kv2.1 T1 domains. Functional Kv channels are tetramers, and we asked whether these two motifs could still be involved in stabilizing either homotetrameric or heterotetrameric assemblies of Kv2.1 T1 by examining the interface of models generated using *YASARA* or *AlphaFold*2. It is likely that Zn^2+^ binding increases the stability of all forms, while the CDD motif should make Kv2.1 homodimers or homotetramers more compact and potentially more stable (Supplementary Fig. S2).

### 3.2. Kv2.1 T1 forms a pentameric conformation in solution

**Figure 4:**
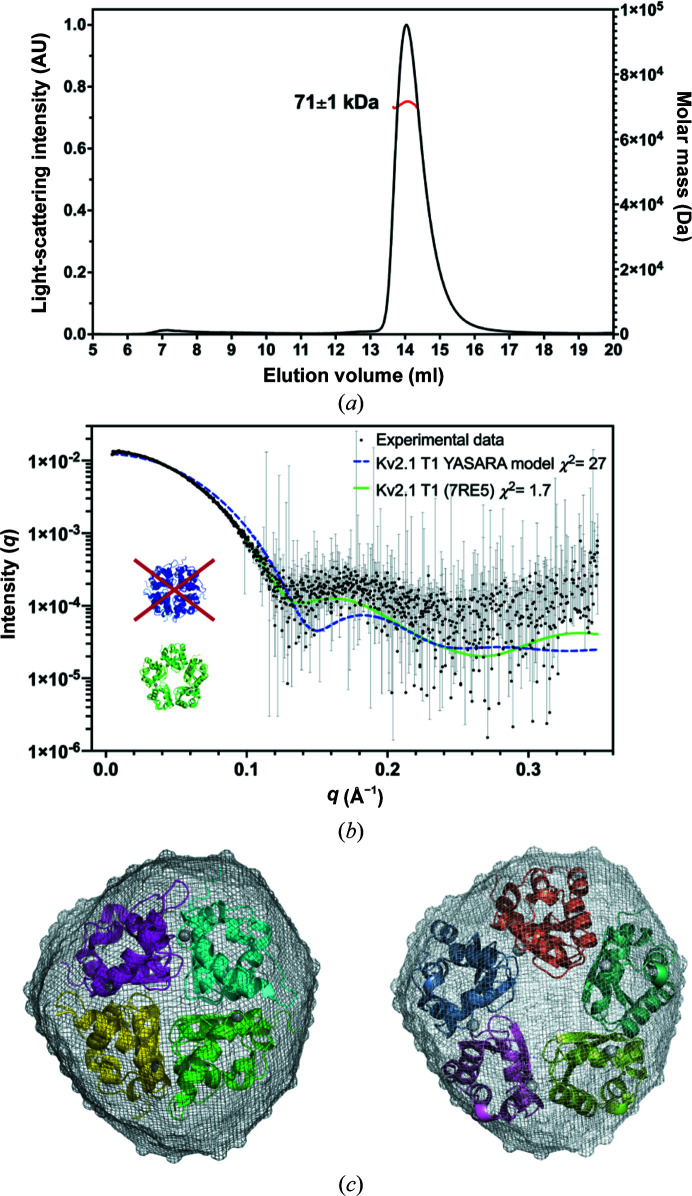
MALS–SAXS analysis of Kv2.1 T1. (*a*) MALS analysis of Kv2.1 T1. The calculated molecular weight (mean ± SD) is taken from the portion of the light-scattering peak indicated in red. (*b*) The experimental scattering curve of Kv2.1 T1 (black) is compared with theoretical scattering curves generated by *CRYSOL* for a tetrameric homology model of Kv2.1 T1 generated using *YASARA* based on Kv3.1 T1 (PDB entry 3kvt, blue, χ^2^ = 27) and the pentameric Kv2.1 T1 crystal structure (green, χ^2^ = 1.7). (*c*) The *ab initio* model of Kv2.1 T1 (shown as a surface) was reconstructed using *GASBOR* and the averaged filtered shape from *DAMFILT* is shown. The model is superimposed with cartoons for the tetrameric Kv2.1 T1 *YASARA* homology model (left) and the pentameric Kv2.1 T1 crystal structure (PDB entry 7re5; right).

**Figure 5:**
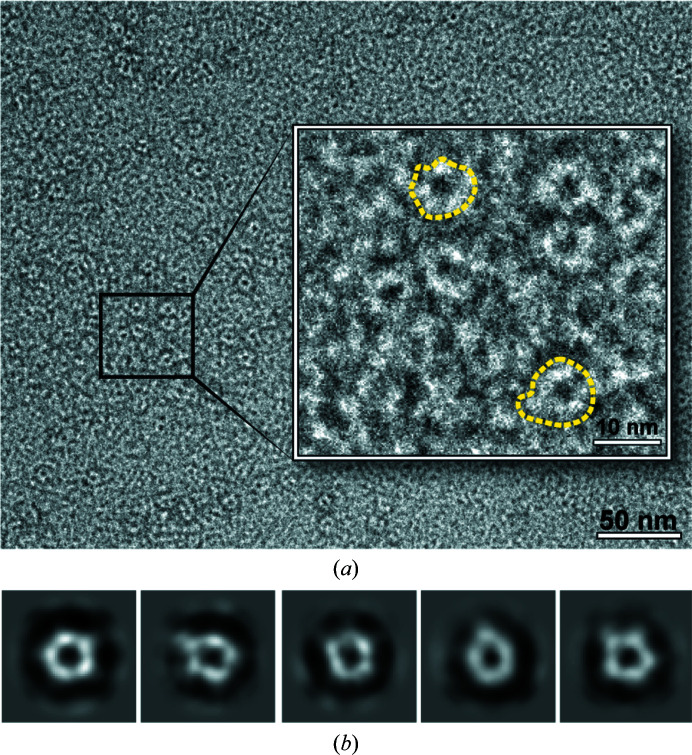
Negative-stain transmission electron microscopy of Kv2.1 T1. (*a*) An electron micrograph of a Kv2.1 T1 sample negatively stained with 2%(*w*/*v*) uranyl acetate and imaged at 100 000× magnification. Inset: increased magnification to aid the visualization of individual oligomers; two examples are outlined with yellow dashed lines. (*b*) 2D reference-free representative class averages of particles in a top view revealed a pentameric form for Kv2.1 T1.

We used multi-angle light scattering and small-angle X-ray scattering coupled with size-exclusion chromatography (SEC–MALS–SAXS) as an independent method to determine the stoichiometry of Kv2.1 T1 complexes in solution. The molecular weight of the Kv2.1 T1 monomer is 14.6 kDa, so the theoretical mass of a tetramer is 58.4 kDa, while that of a pentamer is 73 kDa. The absolute molecular mass of Kv2.1 T1 measured by MALS was 71 ± 1 kDa (Fig. 4 ▸ *a*). The MALS data are most consistent with a pentameric assembly of Kv2.1 T1. From the SAXS data, it was determined that the Kv2.1 T1 protein was monodisperse (linear in a Guinier plot) and globular (with a Gaussian peak in a Kratky plot) (Supplementary Fig. S3). The experimental scattering curve of Kv2.1 T1 was fitted to a tetrameric homology model of Kv2.1 T1 generated using *YASARA* (based on Kv3.1 T1; PDB entry 3kvt), but the fit was very poor, with χ^2^ = 27. Fitting the experimental scattering curve to the pentameric crystal structure resulted in a good fit with χ^2^ = 1.7 (Fig. 4 ▸ *b*). To aid in visualization, the calculated surface envelope for Kv2.1 T1 was superimposed with cartoons for the tetrameric homology model or pentameric crystal structure (Fig. 4 ▸ *c*). The pentameric structure best fits the SAXS data. We also used negative-stain electron microscopy (EM) to investigate the oligomeric state of Kv2.1 T1. Top views of ring-shaped particles for Kv2.1 T1 could be seen in the raw electron micrographs, and after 2D class averaging the rings could be more clearly seen to be pentamers (Fig. 5 ▸). In conclusion, analysis of Kv2.1 T1 in solution by SEC–MALS–SAXS complemented with direct visualization by EM is consistent with the pentameric assembly revealed by the crystal structure.

### 3.3. The effect of bound zinc on the stability of Kv2.1 T1

**Figure 6:**
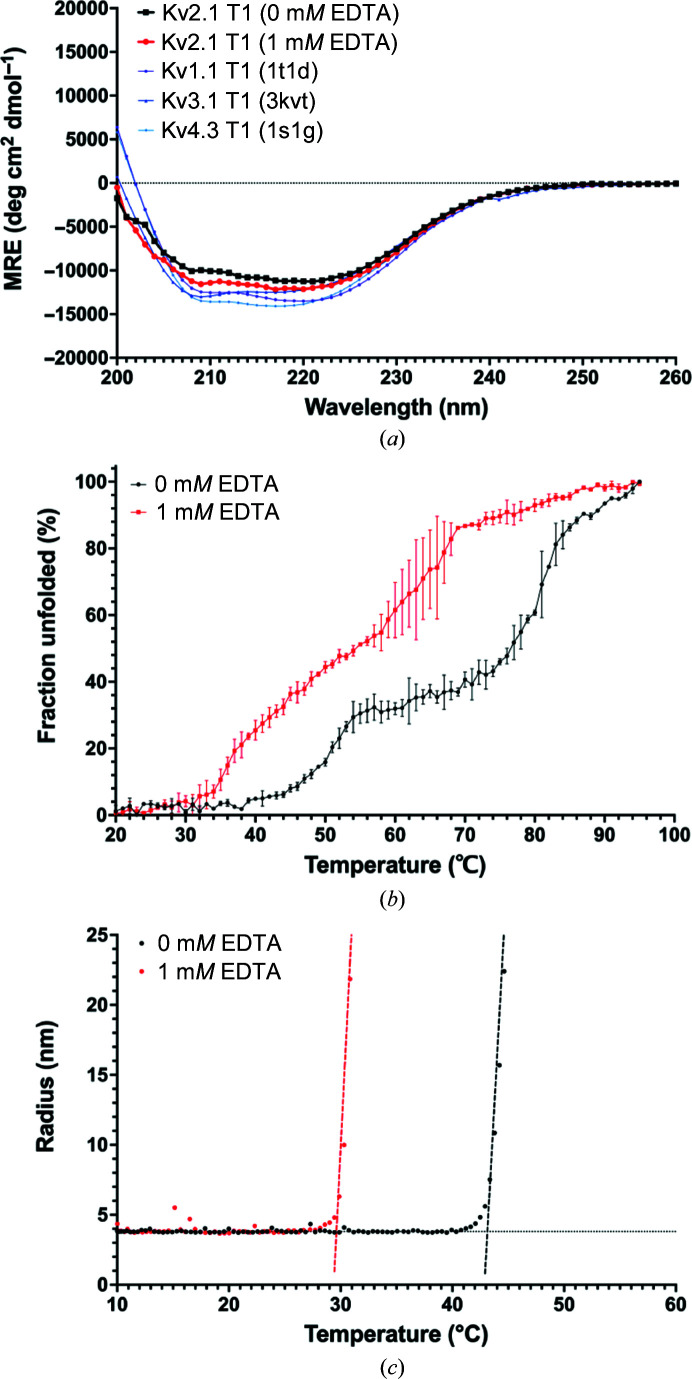
Protein thermal unfolding in the presence of EDTA. (*a*) Comparison of CD spectra of Kv2.1 T1 at 10°C in the absence (black) or presence (red) of 1 m*M* EDTA with the calculated spectra for Kv1.1 (PDB entry 1t1d, purple), Kv3.1 (PDB entry 3kvt, blue) and Kv4.3 (PDB entry 1s1g, light blue) T1 domains. (*b*) Stability analysis of Kv2.1 T1 in solution using CD. The fraction of unfolding extracted from far-UV CD spectra at 222 nm was plotted as a function of temperature. (*c*) The hydrodynamic radius of Kv2.1 T1 monitored by dynamic light scattering is presented as a function of temperature. In (*b*) and (*c*), the black data points are for Kv2.1 T1 without EDTA and the red data points are for Kv2.1 T1 treated with 1 m*M* EDTA.

The role of Zn^2+^ in Kv3 and Kv4 T1 domains has been determined to be critical for the transition from monomers to tetramers as well as for protein stability (Jahng *et al.*, 2002 ▸; Strang *et al.*, 2003 ▸). To investigate the effect of bound Zn^2+^ on the assembly and stability of Kv2.1 T1, we used chelation by excess EDTA (1 m*M*) and assessed the effect that this had on the protein using circular-dichroism (CD) and dynamic light-scattering (DLS) thermal unfolding experiments. The CD spectra calculated for Kv1.1, Kv3.1 and Kv4.1 T1 domains determined using *PDB*2*CD* (Mavridis & Janes, 2017 ▸) was very similar to the experimental spectra obtained for Kv2.1 T1. A strong α-helical signal for Kv2.1 T1 persisted at 10°C after the addition of EDTA, implying that Kv2.1 T1 remains in a native fold in this temperature range independent of bound Zn^2+^ (Fig. 6 ▸ *a*).

The thermal unfolding experiments were monitored by CD at 222 nm at 1°C intervals from 20 to 95°C (Fig. 6 ▸ *b*). The fraction of unfolded protein (fU) as a function of temperature exhibited two transitions. The α-helical signal began to diminish at 40°C and unfolding increased until ∼60°C, and at ∼80°C the amount of unfolded protein again increased. In the presence of EDTA the curve was shifted to the left and the differences between the two phases were less striking. To calculate the melting temperature (*T*_m_, defined as fU = 0.5), the fraction of unfolded protein (fU) as a function of temperature from 10 to 70 °C (to encompass the first transition) was fitted by a sigmoidal curve. The *T*_m_ of Kv2.1 T1 was 50 ± 1°C and decreased to 38 ± 1°C in the presence of EDTA. Our results imply that Kv2.1 T1 undergoes a large conformational change at lower temperature in the absence of bound Zn^2+^. Using DLS as a complementary approach, we monitored the hydrodynamic radius as a function of temperature (10–80°C). The onset transition temperature (or the threshold where protein unfolding begins; *T*_onset_) of Kv2.1 T1, defined by a linear transition plot, was 42.5 ± 0.5°C. Addition of EDTA reduced the *T*_onset_ to 29.5 ± 0.5°C (Fig. 6 ▸ *c*). These data support the CD experiments and we conclude that Zn^2+^ binding increases the stability of the protein.

## 4. Discussion

Here, we report the crystal structure of the T1 domain of Kv2.1. This T1 domain is very similar to previously described T1 domains from other Kv subfamilies, except that it forms a pentamer. All of the previously reported structures of isolated Kv T1 domains are tetramers, which is consistent with the tetrameric assembly of full-length Kv subunits into functional channels. Our X-ray structural data are supported by solution analysis of the Kv2.1 T1 domain using SEC–MALS–SAXS and EM imaging.

In the larger family of BTB folds (of which the T1 domain is one class), there is a variety of stoichiometries from monomers to hexamers (Stogios *et al.*, 2005 ▸; Sereikaite *et al.*, 2019 ▸). Illustrative to Kv2.1 T1 are the T1-like domains from the Cullin-dependent ubiquitin E3-ligases, where the stoichiometry is particularly diverse. In one study, the T1-like domains of SHKBP1, KCTD13, KCTD16 and KCTD17 crystallized as a monomer, a tetramer, an open pentamer and a closed pentamer, respectively, even though most of these T1-like domains are pentamers in the context of the full-length native protein. In solution, the T1-like domains of SHKBP1 and KCTD13 were predominantly mixtures of monomers and dimers, but mixing with the binding partner Cullin3 induced pentamers (Pinkas *et al.*, 2017 ▸).

We assume that the pentameric state that we observed for Kv2.1 T1 represents either an intermediate state that is possible as Kv2.1 monomers assemble into functional, tetrameric channels or a simple epiphenomenon of working with the T1 domain out of the context of the full-length channel. We propose that for full-length native Kv2.1 the T1-domain assembly state is restrained to a tetramer by interactions with either other parts of the channel or accessory subunits. In support of this idea, interactions between the cytoplasmic N-termini and C-termini of Kv2 have been observed (Ju *et al.*, 2003 ▸; Mohapatra *et al.*, 2008 ▸; Bocksteins *et al.*, 2009 ▸). Intriguingly, a recent structural study discovered that the orientation of the T1 domain in Kv3 channels differs from that in structures of the Kv1.2–2.1 paddle chimera. The most C-terminal α-helix of the Kv3.1a T1 domain is rotated towards and contacts the linker between the voltage-sensor and pore domains, which may stabilize the pore and contribute to the unique kinetic properties of Kv3 channels (Chi *et al.*, 2021 ▸). It remains to be determined whether a similar arrangement occurs in Kv2 channels.

In terms of accessory subunits, the Kvβ subunit is best characterized for its interaction with Kv1 (Shaker) channels, and several KChIP proteins serve a similar role to Kvβ for Kv4 channels (Pongs *et al.*, 1999 ▸; Bähring, 2018 ▸). Both Kvβ and KChIP proteins make contacts with the T1 domain of the cognate Kv protein and in the case of KChIP can even rescue mutants of Kv4 T1 domains that interfere with tetramerization (Kunjilwar *et al.*, 2004 ▸; Liang *et al.*, 2010 ▸). For Kv2.1 the best characterized interaction proteins are the electrically silent (KvS) α subunits (Bocksteins, 2016 ▸) and those mediating the plasma membrane–ER contact sites organized by phosphorylated Kv2.1 in a scaffolding role independent of ion conduction (Deutsch *et al.*, 2012 ▸; Weigel *et al.*, 2012 ▸; Fox *et al.*, 2013 ▸, 2015 ▸; Johnson *et al.*, 2018 ▸; Antonucci *et al.*, 2001 ▸; Misonou *et al.*, 2004 ▸; Kirmiz, Palacio *et al.*, 2018 ▸; Kirmiz, Vierra *et al.*, 2018 ▸). The recent acceleration in solving structures of ion channels, with and without accessory proteins, by cryo-EM makes it reasonable to expect that a structure of full-length Kv2.1 will be determined and provide information about the interactions that influence the T1 domain.

The interface between Kv2.1 T1 subunits in the pentameric ring involves two major features: electrostatics and inter-subunit coordination of zinc. At the N-terminal end of the domain there is a large highly conserved patch of surface negative charges that fits into a concavity of positive charges on the adjacent subunit. This is also where the CDD motif is found, a motif that is recognized for its selective conservation in Kv2 and KvS proteins (Bocksteins *et al.*, 2009 ▸). Charge-reversal mutants of the aspartates in the CDD motif interfered with the assembly of Kv2.1. The original homology modeling of Kv2.1 T1 positioned the CDD residues away from the T1 interface, so that the assembly problem was thought to have been due to disruption of an interaction with the C-terminus (Mohapatra *et al.*, 2008 ▸). However, in our structure the aspartates forming the CDD motif participate in a salt bridge with two arginines at the N-terminus of T1 which contributes to stabilizing the T1–T1 interface. Caution must be employed because this salt bridge may be disrupted in the physiologically relevant tetrameric form of the Kv2.1 T1–T1 interface, although our modeling predicts that this is only true for heterotetrameric assemblies of Kv2.1 with KvS T1 domains.

Inter-subunit coordination of zinc by Kv3 and Kv4 T1 domains has been shown to be critical for the stability of the protein as well as for the assembly of tetramers. Kv4.2 T1 is a particularly interesting case because chelation with EDTA to remove zinc has been shown to convert tetramers to monomers, which could reform into tetramers upon the replacement of zinc (Jahng *et al.*, 2002 ▸). For Kv2.1 T1 we obtained two structures, one with full zinc occupancy and one with partial zinc occupancy; in both structures the T1 domain was a pentamer. This indicates that zinc binding alone may not be sufficient to dictate the stoichiometry of the Kv2.1 T1 domain. Using CD and DLS, we were able to show that removing zinc by EDTA chelation reduced the stability of the Kv2.1 T1 protein; this latter observation is consistent with the role of zinc binding in stabilizing Kv3 and Kv4 T1 domains.

Our observations of the pentameric Kv2.1 T1–T1 interface have implications for understanding why only T1 domains from Kv2.1 or Kv2.2 can interact with T1 domains from the ten-member KvS family. Four of the KvS T1 domains (Kv8.2, Kv6.1, Kv6.2 and Kv6.3) appear to have lost the ability to bind zinc since the histidine in the H*X*_5_C*X*_20_CC Zn^2+^ coordination motif is not conserved in these proteins. This alteration would not prevent these T1 domains from providing the cysteine needed to coordinate the Zn^2+^ ion bound in an adjacent Kv2.1 T1 domain, but it would result in only partial zinc occupancy of the tetrameric channels. Since partial zinc occupancy did not prevent the crystallization of a Kv2.1 homopentamer, it is possible that partial zinc occupancy is not as deleterious to Kv2.1-containing channels as it is predicted to be for Kv3- or Kv4-containing channels. This could explain why at least four KvS T1 domains do not assemble with Kv3 or Kv4 T1 domains. A feature that impacts all ten KvS T1 domains is the lack of a pair of arginines to form the salt bridge with the aspartates in the CDD motif. If this salt bridge is important in the tetramer as well as the pentamer that we see here, it would imply that the Kv2 and KvS proteins would be most stable in a 3:1 stoichiometry. Both 2:2 and 3:1 stoichiometries have been observed for Kv2 and KvS proteins in transfected cells, but the state that predominates in native channels is unknown (Pisupati *et al.*, 2018 ▸, 2020 ▸; Möller *et al.*, 2020 ▸; Kerschensteiner *et al.*, 2005 ▸).

In conclusion, we provide the first structural analysis of the human Kv2.1 T1 domain. Since structures of Kv1, Kv3 and Kv4 T1 domains have been solved, we started this work with the expectation of providing a representative T1 domain for the missing channel-forming Kv subfamily. We were consequently surprised to find that the Kv2.1 T1 domain behaves differently from the others in the stoichiometry that we observed. Future work will need to determine how the Kv2.1 T1 domain is arranged in the context of the full-length channel. While this manuscript was under review, an abstract was published indicating that a cryo-EM structure of full-length Kv2.1 will soon be available for just such an analysis (Fernández-Mariño *et al.*, 2022 ▸).

## Supplementary Material

PDB reference: Kv2.1 tetramerization domain, 7re5

PDB reference: 7spd

Supplementary Figures and Table. DOI: 10.1107/S205979832200568X/jv5009sup1.pdf

A preprint of this article is available from bioRxiv.: https://doi.org/10.1101/2021.11.09.467962

## Figures and Tables

**Table 1:** Summary of X-ray data-collection and refinement statistics for PDB entry 7re5 Values in parentheses are for the highest resolution shell.

Data collection	
Beamline	Beamline 4.2.2, ALS
Wavelength (Å)	1.072
Space group	*P*4_1_2_1_2
*a*, *b,* *c* (Å)	78.75, 78.75, 214.80
α, β, γ (°)	90, 90, 90
Resolution (Å)	44.37–2.50 (2.59–2.50)
Total reflections	48604 (4776)
Unique reflections	24302 (2388)
Multiplicity	2.0 (2.0)
Completeness (%)	99.93 (99.92)
Mean *I*/σ(*I*)	27.98 (2.45)
Wilson *B* factor (Å^2^)	48.02
*R*_merge_	0.02631 (0.2805)
*R*_meas_	0.03721 (0.3967)
*R*_p.i.m._	0.02631 (0.2805)
CC_1/2_	0.999 (0.856)
CC*	1 (0.961)
Refinement	
Reflections used in refinement	24293 (2387)
Reflections used for *R*_free_	1207 (111)
*R*_work_	0.2250 (0.3135)
*R*_free_	0.2673 (0.3824)
No. of non-H atoms	
Total	4349
Macromolecules	4316
Ligands	8
Solvent	25
Protein residues	542
R.m.s.d, bond lengths (Å)	0.01
R.m.s.d, angles (°)	1.2
Ramachandran favored (%)	98.50
Ramachandran allowed (%)	1.50
Ramachandran outliers (%)	0.00
Rotamer outliers (%)	3.05
Clashscore	7.04
Average *B* factor (Å^2^)	
Overall	51.89
Macromolecules	51.9
Ligands	69.81
Solvent	46.09
