# Supplementary material for: Pentameric assembly of the Kv2.1 tetramerization domain
Source: Acta Crystallogr D Struct Biol. 2022 May 30;78(Pt 6):792–802. doi: 10.1107/S205979832200568X (PMC9159280; doi:10.1107/S205979832200568X)
Supplement: Supplementary file 1 [file d-78-00792-sup1.pdf]

STRUCTURAL BIOLOGY

Volume 78 (2022)

Supporting information for article:

Pentameric assembly of the Kv2.1 tetramerization domain

Zhen Xu, Saif Khan, Nicholas Schnicker and Sheila Baker

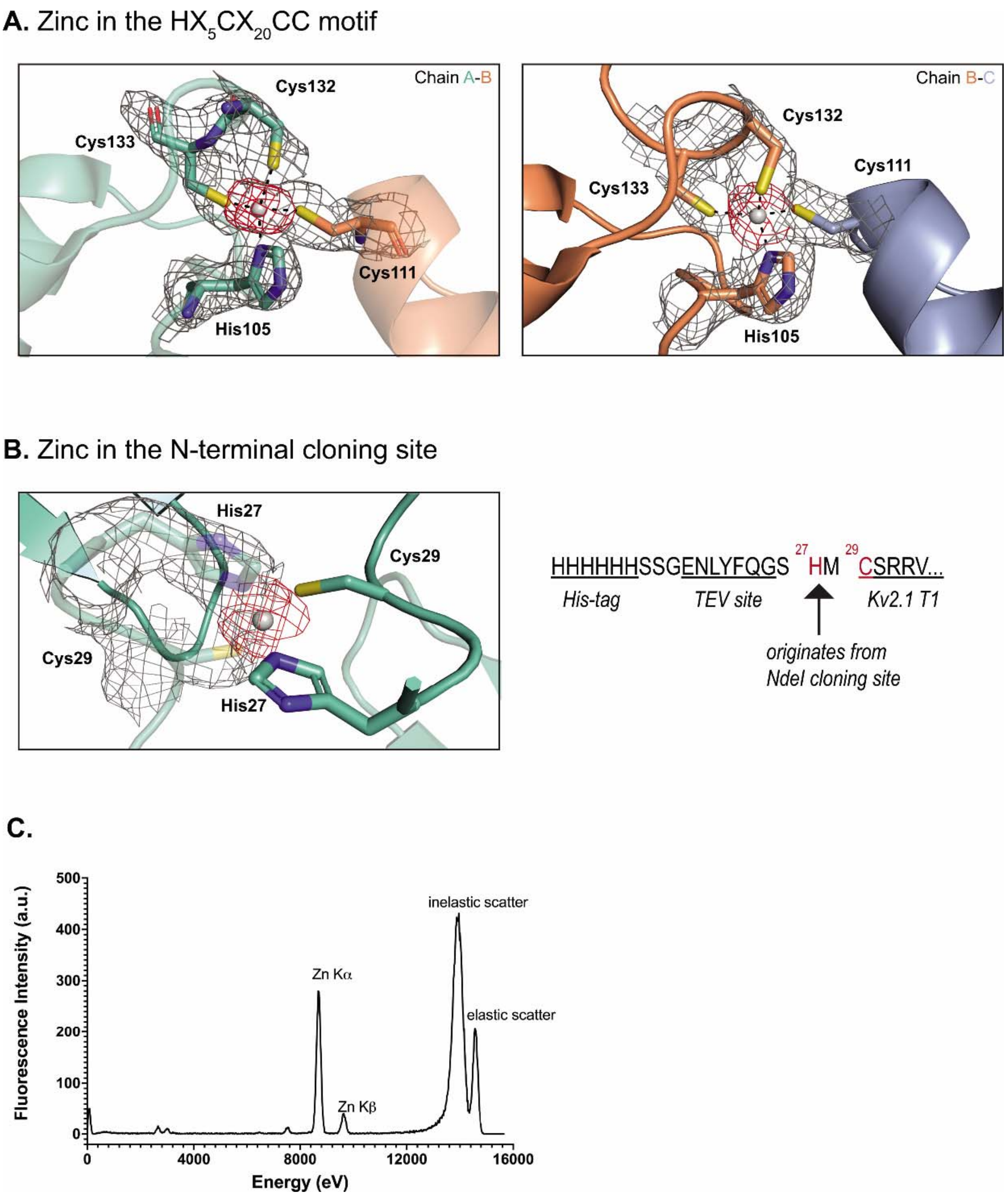

Figure S1 Overview of Zn^{2+} coordination in Kv2.1 T1. **(A)** Tetrahedral coordination of Zn^{2+} by the HX$_5$CX$_{20}$CC motif. *Left*, the electron densities for Zn^{2+} interaction with His105, Cys132 and Cys133 from chain A and Cys111 from chain B. *Right*, the electron densities for Zn^{2+} interaction with His105, Cys132 and Cys133 from chain B and Cys111 from chain C. Residues are shown in stick representation, zinc ion in gray, sulfide in yellow, nitrogen in dark blue. Coordination bonds are marked with black dashed lines. The gray grid represents the 2mFo – DFc map (1.0σ); red, anomalous map (3.0σ) **(B)** Tetrahedral coordination of Zn^{2+} with His27 (introduced by the NdeI cloning site) and Cys29 from chain A and its symmetry mate in crystal packing. **(C)** Energy spectrum obtained from X-ray fluorescence measurement of the crystal indicates the presence of zinc. The zinc Kα peak is located at 8.6 keV and the zinc Kβ peak is located at 9.6 keV.

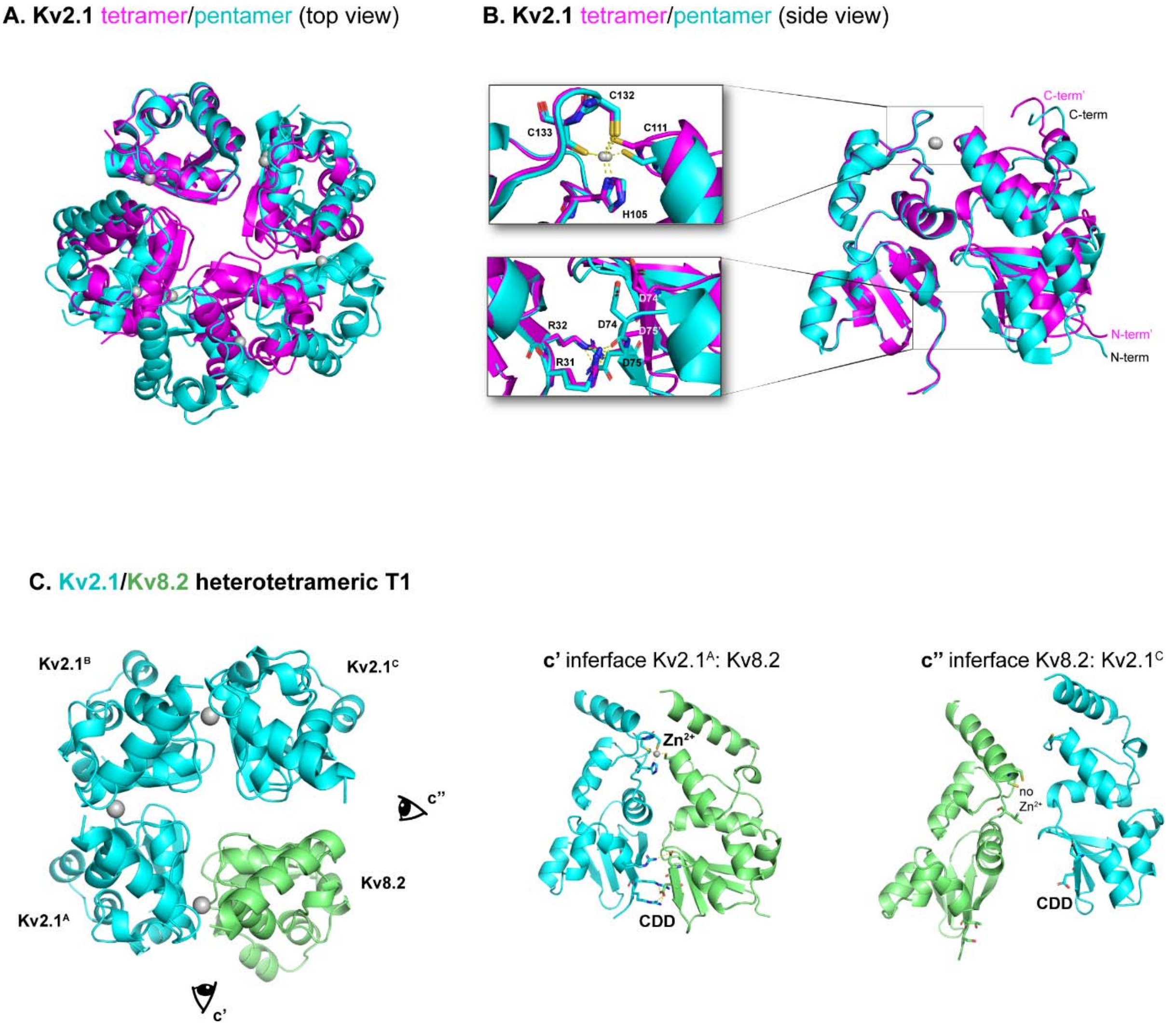

Figure S2 Comparison of the pentameric Kv2.1 T1 crystal structure to models of homotetrameric and heterotetrameric Kv2.1 T1. **(A)** Overlay of the homopentameric Kv2.1 T1 crystal structure (in cyan, top view as in Figure 1A) on a homotetrameric model of Kv2.1 T1 (in magenta). The homotetrameric model was generated using the monomer from our crystal structure (PDB: 7RE5) superimposed on Kv4.2 T1 (PDB: 1NN7) and YASARA was used for energy minimization; no major clashes occurred. **(B)** Predicted interface of Kv2.1 T1-Kv2.1 T1 from the homotetrameric model, with enlarged views of the Zn^{2+} coordination and CDD motifs in the insets. These motifs are likely to contribute to the stability of tetrameric assemblies. **(C)** Model of a 3:1 Kv2.1: Kv8.2 T1 heterotetrameric complex generated using AlphaFold2 (Kv2.1 is shown in cyan, Kv8.2 in green). **(c' and c'')** Side views of the two different Kv2.1: Kv8.2 T1 interfaces. Note, Kv8.2 does not bind Zn^{2+} and the lack of a salt bridge formed by the Kv2.1 CDD introduces a large gap in the interface.

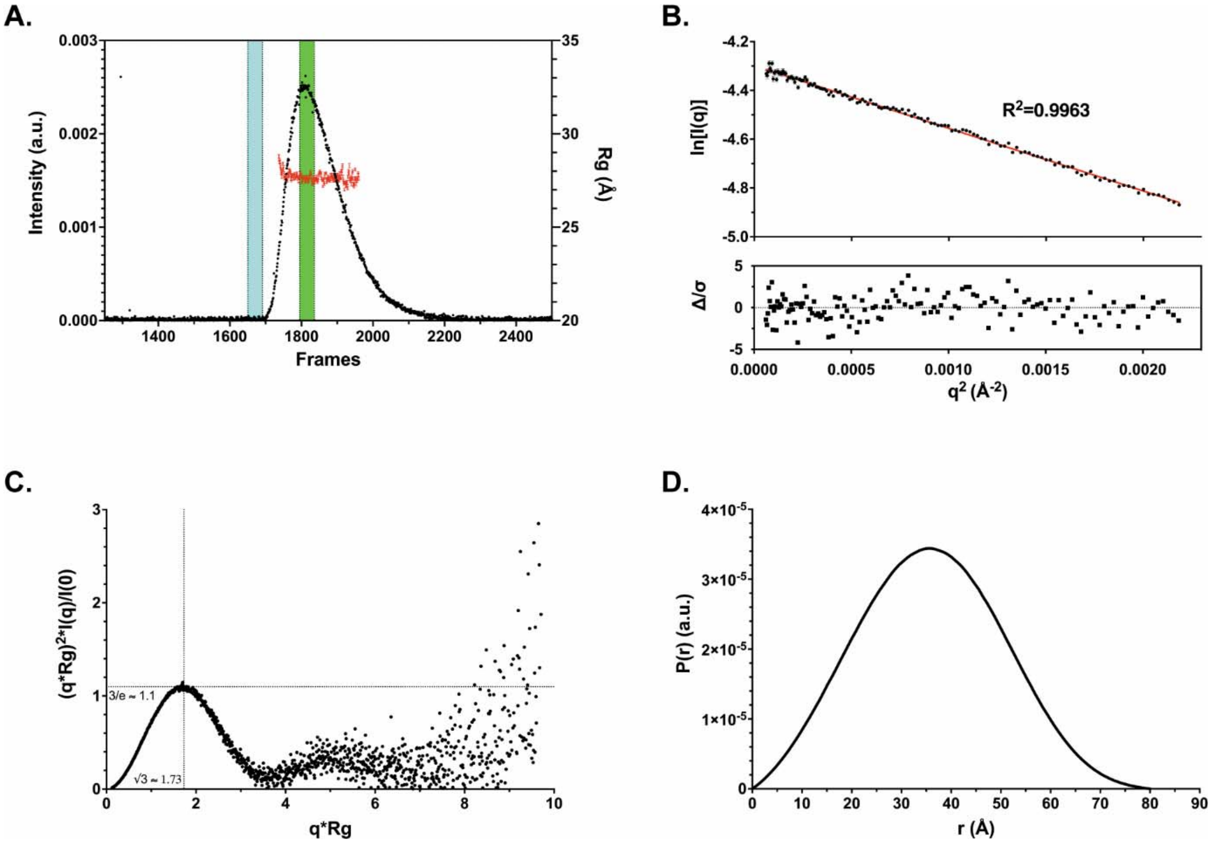

Figure S3 Analysis of Kv2.1 T1 SAXS data quality. (**A**) The total scattering intensity aligns with the Rg plot of Kv2.1 T1 in-line SEC-SAXS frames. The data in the buffer region (cyan) and the sample region (green) were used for buffer subtraction and data analysis. **(B)** The linear low-q region (qRg < 1.3) of the scattering curve was used in Guinier analysis. The Rg of Kv2.1 T1 was 27.7 ± 0.1 Å. **(C)** Rg normalized dimensionless Kratky analysis indicates Kv2.1 T1 is well folded in solution. **(D)** Pair distance distribution P(r) function suggests the Dmax of Kv2.1 T1 was 80 Å.

Table S1 SAXS data collection and analysis parameters

**a) Sample details**	
SEC Column	Superdex 200 increase 10/300
Loaded concentration (mg/ml)	1.5
Injection volume (ul)	250
Flow rate (ml/min)	0.5
Solvent (solvent blanks taken from SEC flowthrough prior to elution of protein)	20 mM Tris-HCl pH 8.0, 150 mM NaCl, 5% glycerol, 0.01% Triton X-100, 10 mM BME
**b) SAXS data-collection parameters**	
Instrument	BioCAT facility at the Advanced Photon Source beamline 18ID with Pilatus3 1M (Dectris) detector
Wavelength (Å)	1.033
Beam size (um2)	150 (h) x 80 (v)
Camera length (m)	3.5
q measurement range (Å-1)	0.0045-0.35
Absolute scaling method	N/A
Basis for normalization to constant counts	To incident intensity, by ion chamber counter
Monitoring for radiation damage	Frame-by-frame comparison of data
Exposure time	0.5 s exposure time with 1 s total exposure period of entire SEC elution
Sample configuration	SEC-MALS-DLS-RI-SAXS. Size separation used a Superdex 200 increase 10/300 GL column and an Infinity II HPLC (Agilent Technologies). Flow was in line with the UV-MALS-DLS-RI instruments and SAXS after the column. UV data was measured in the Agilent, and MALS-DLS-RI data by DAWN HELEOS-II (17 MALS + 1 DLS channels) and Optilab T-rEX (RI) instruments (Wyatt). SAXS data was measured with sheath-flow cell in a 1.5 mm ID 1.52 mm OD quartz capillary, effective path length 0.49 mm.
Sample temperature (ºC)	22
**c) Software employed for SAXS data reduction, analysis, and interpretation**	

SAXS data reduction	Radial averaging, background subtraction, frame comparison, averaging, and subtraction were made using BioXTAS RAW 2.0.2.
Basic analysis: Guinier, MW, Normalized Kratky, P(r)	Guinier plot and molecular weights were calculated using BioXTAS RAW 2.0.2, P(r) function using GNOM from ATSAS 3.0.
MALS-DLS-RI analysis	Astra 7.1.3 (Wyatt)
**d) Structural parameters**	
Guinier analysis	
R_g (Å)	27.7 ± 0.1
q-range (Å-1)	0.00786-0.04587
$q$maxR_g	1.272
R_g (Å)**	27.3
Dmax ((Å)**	80
Volume (Å3, adjusted V_P as SAXS MoW2)	88300
MW, Vp method (kDa)	73.3
MW, MALS (kDa)	71 ± 1
MW, Theoretical by sequence (kDa)	73.1
